# Why Do People Migrate? Fresh Takes on the Foundational Question of Migration Studies

**DOI:** 10.1177/01979183241269445

**Published:** 2024-09-02

**Authors:** Jørgen Carling

**Affiliations:** Peace Research Institute Oslo (PRIO), Oslo, Norway

**Keywords:** migration theory, motivations for migration, causes of migration

## Abstract

“Why do people migrate?” is a question that forms the pivot of migration studies, and migration theory in particular. But it has hardly found satisfactory answers. In this article, I reapproach the question from an array of diverse angles and provide eight responses. Some are aligned with recent theoretical developments, others unpack long-standing ideas with evolving significance, and still others are fundamentally atheoretical. Together, they show how the question can be answered, how it is being answered—even inadvertently or misleadingly—and what the implications are of answering the question in different ways. These are the responses, which each initiates a discussion: (1) For the reasons under which they are admitted as immigrants; (2) For reasons that are socially legitimate; (3) Because the sum of push and pull factors is in favor of migration; (4) Because they have the aspiration and the ability to do so; (5) Because an opportunity presents itself; (6) Either because they chose to or because they are forced to; (7) Because they see migration as either intrinsically or instrumentally valuable; (8) To lead a normal life. The discussions demonstrate how theoretical, methodological and political dimensions of migration sway the ways in which reasons for migration are understood and represented. “Why do people migrate?” is slippery as a research question, but its indeterminate nature makes it a guiding light for research that navigates a diversity of perspectives with humility and curiosity.

## Introduction

Few questions are as foundational to migration studies as “why do people migrate?” Yet the most common answer is banal: for many different reasons. In this article I reapproach the question from eight diverse angles. Asking why people migrate does not call for an index of reasons, but for a reflexive inquiry into the various ways in which this question might be meaningfully answered.

While the question “why do people migrate?” is a staple of introductory courses, it has been met with skepticism in the scientific literature. In a recent influential article, Hein de Haas (2021) argues that “the relevant theoretical question is […] not ‘why people move’ (which tends to yield overly generic and rather meaningless platitudes of the ‘push–pull’ genre) but, rather, how patterns and experiences of migration are shaped by broader processes of social change.” And Ronald Skeldon, who formulated the original dismissal of push–pull theory as “a platitude as best” (1990, 126), has made the broader claim that “‘why?’ is not a scientific question.”^1^

The skepticism from leading scholars is understandable, but should not make us dismiss “why do people migrate?” as a starting point for inquiry. Although the question is only rarely tackled head-on by migration researchers, it permeates the categories and concepts that are used, and the way analyses are designed. In other words, the literature is fertile ground for examining and synthesizing the diversity of oblique approaches to a question that seems, at once, trite and impossibly profound.

The “why” of migration figures more unabashedly in bureaucracy, popular culture, and everyday life. In these spheres, too, it inspires contemplation on how and why the question is asked and answered. “Why did you come to the United States?” is the first item on the intake questionnaire for unaccompanied child migrants that the author Valeria Luiselli used as an interpreter in a federal immigration court. In the book that she subsequently wrote about this experience, Luiselli (2017, 9) turns the question onto herself and her Mexican family. “We didn’t have a clear answer” she asserts. “No one ever does.” But the book ends with a lucid answer, given by a little girl among the unaccompanied minors. When Luiselli asks why she came, the girl asserts “Because I wanted to arrive.”

For many migrants, it is, in fact, the opposite. Migration is often about leaving. “Why did you leave Colombia?” asks the protagonist of Patricia Engel's (2023, 189) short story *Aguacero*. “The same reason everyone leaves” her interlocutor answers. “Colombia is a rabid dog.” This evocative response reflects the simultaneously burning and elusive motivations that migration researchers have explored through fieldwork in communities of origin. Ethnographers working in North Africa and the Balkans, for instance, have encountered a deep sense of “futurelessness” or “intensity of everyday despair” among young adults that motivates migration (e.g., Anđić 2020b; Manolova 2019; Schielke 2008; Souiah 2012). Similarly, one of Maddy Thompson's (2017, 81) interviewees in the Philippines said that “I really see how hopeless this country is. That's why I realised that I have to get out.”

In parallel with ethnographic studies, survey researchers have struggled to develop meaningful questions, response options, and analytical strategies for understanding and quantifying why people migrate. In an enlightening methodological paper on survey data, Gillespie, Mulder, and Eggleston (2021, 2), conclude that “it is no simple task for migration researchers to collect the information on migration motives that they seek.”

Migration is a “demographic event,” an action that people take, which we may want to understand. But it is also a process and a lasting state. Extending the timeframe opens up new questions: Why is this person still a migrant? How does she identify with her original reasons for migration? How have those reasons—or how they are represented—changed over time? Such explicit engagement with temporal dimensions has become central to migration studies over the past decade. It falls outside the scope of this article, but stands as inspiration for further enquiry of the issues that I raise.

The bulk of this article is structured around eight different responses to the question “why do people migrate?” Thereafter, I provide a brief summary overview of the functions and implications of each response and discuss ways in which “whys” can cascade in sequential steps. The concluding section grounds the insights in issues related to methodology and research practice.

## Why Do People Migrate?

The answers I put forth over the following pages are starting points for reflection and discussion, and they have been selected for this purpose. Some are aligned with recent theoretical developments, others unpack long-standing ideas with evolving significance, and still others are fundamentally atheoretical. Together, they show how the question *can be answered*, how it *is being answered*, even inadvertently or misleadingly, and the promises and pitfalls of answering the question in different ways.

### 1. For the Reasons Under Which They Are Admitted as Immigrants

Immigrants are admitted as workers, family members, students, or refugees, and such groupings pervade more general thinking about reasons for migration. As Cecilia Menjívar (2023, 9) observes, state categories are not only *constructed* entities, but tend to become *normalized*: “People absorb official classifications into frames through which they make sense of the world and those around them.” Beyond the realm of government and official statistics, the categories of immigrant admission are prominent organizing principles in migration scholarship. However, the connection between the policy logic and migrant motivations is tenuous.

The first point to note is how government practice equates the administrative categories with reason for immigration. In several European countries, official statistics classify immigrants in such a way. They typically differentiate between four main reasons: work, family, education, and protection (or humanitarian). In Norway, such numbers are unreservedly published with the title “reasons for immigration,” based on the type of permit under which each person was admitted. This exemplifies the equation of administrative categories with migration motives in its most naïve form.

With greater nuance, French authorities publish numbers of “residence permits by motive,” by which they strictly speaking mean motive for giving the permit. But even in analyses produced by the statistical institute's own staff, the same data figure as “motive for migration” (Akgüç and Welter-Médée 2021).

Then there are statistics that appear similar—allocating immigrants to categories such as family, labor, and education—but are produced by a different route. Dutch authorities assign a so-called “derived migration purpose” based on the country's detailed social statistics. For instance, the “labor” purpose is applied to any immigrant who, within 120 days of their entry, gets the bulk of their income from labor. What is striking here, is that even when it is not necessary or possible to classify on the basis of permits, the categories as such are upheld. The richer information is simply used to place individuals in their “correct” category.^2^

An obvious problem with deducing motivations from types of permits is that migrants may choose the immigration path of least resistance, regardless of their primary motivation. For instance, refugees who languish in camps with improbable hopes of resettlement might seek alternative pathways based on educational or labor migration (Aden 2023; Jayasuriya 2016; Poole and Riggan 2020). Similarly, binational couples might weigh the pros and cons of securing joint residence based on employment or marriage. Research on student migration, too, has highlighted the separate roles of education-related motivations and mobility-enabling student visas (Adeyanju and Olatunji 2021; Nyamnjoh 2021).

A rare empirical demonstration of this point comes from the THEMIS survey, covering Brazilian, Moroccan, and Ukrainian migrants in four European countries. Table 1 shows the respondents’ most important motivation for coming to the destination country, by the first type of residence permit they held.

**Table 1.** Permit Types and Most Important Migration Motivation, THEMIS Survey (%).

	Type of residence permit held		
Most important motivation	Employment	Family	Education
Opportunities for work	70	19	15
Being with specific people	11	67	6
Opportunities for study	5	6	58
Experiencing another culture	12	6	12
Learning a language	2	2	9
Total	100	100	100

*Note*: Immigrants from Brazil, Morocco, and Ukraine residing in the Netherlands, Norway, Portugal, and the United Kingdom, interviewed in 2012. Not including less common or unknown permit types. *N* = 1575; weighted to reflect sampling design. See Carling and Jolivet (2016) for details.

The main motivation for migration corresponds to the permit type in most cases, but far from all. The proportion is 70% for those with an employment-based permit, 67% for family-based permit holders, and 58% for students. The remainder, who very roughly make up one-third of the sample, have a different motivation from what their permit would suggest. The most common examples are migrants on family or education permits who are motivated by opportunities for work, and migrants on employment and education permits who are motivated by experiencing another culture. The figures illustrate the risk of conflating categories of analysis with categories of practice (cf. Raghuram 2021).

A second objection to the conventional migration categories is that they are not exhaustive. Even if labor migrants, family migrants, international students, and humanitarian migrants are seen as workable categories, there might be other migrants who don’t fit any of these four. The most prominent addition to the list is *lifestyle migration*, which has inspired research that offers new and illuminating set of answers to the question “why do people migrate?” (Benson and O’Reilly 2016; McGarrigle 2022). Research on retirement migration and return migration has similarly diversified ideas about reasons for migration (Hagan and Wassink 2020; King, Cela, and Fokkema 2021; Pickering et al. 2019; Xiang 2014) Some of this research was spurred by the expansion of free movement within Europe, which created new migration flows that were less reliant on administrative categories (King 2002).

But even with a longer list, it can be constraining to compartmentalize migration into separate types, each defined by a specific motivation. Migration researchers have, of course, pointed out that motivations can be mixed, changing, complex, and conflicting (e.g., Collyer and de Haas 2010). The data from the THEMIS survey again provide an illustration. Table 2 shows *all* important motivations, not only the most important, as in the previous table. We see, for instance, that experiencing another culture is an important motivation for the majority of respondents with all three types of permit. Similarly, opportunities for work motivate the majority of migrants, also those who have a residence permit based on family ties or education. It's also striking that, in just over 10% of the cases, the permit type corresponds to a motivation that was not important at all.

**Table 2.** Permit Types and Important Migration Motivations, THEMIS Survey (%).

	Type of residence permit held		
Important motivations	Employment	Family	Education
Opportunities for work	89	58	51
Being with specific people	37	87	16
Opportunities for study	28	47	89
Experiencing another culture	54	53	79
Learning a language	35	51	61

*Note*: Immigrants from Brazil, Morocco, and Ukraine residing in the Netherlands, Norway, Portugal, and the United Kingdom, interviewed in 2012. Not including less common or unknown permit types. *N* = 1575; weighted to reflect sampling design. See Carling and Jolivet (2016) for details.

What would happen to our understanding of migration motivations in the absence of immigration rules? Research and debate on internal migration give an indication. People also migrate within their own country for reasons that have to do with family, labor, and education, for instance, but are not in the same way portrayed as “family migrants,” for instance (Gillespie, Mulder, and Eggleston 2021). This is yet another reminder to “mind the gap” as King and Skeldon (2010) say, between scholarship on internal and international migration.

Researchers who specifically examine migration motivations typically allow for mixed and non-standard factors (e.g., Luthra, Platt, and Salamońska 2018; Spaan, Henkens, and Kalmijn 2023). But the compartmentalization of the immigration bureaucracy still persists, also in the academic domain. While social science is often reliant on simplification, the simplifications need to be deliberate and analytically sound.

### 2. For Reasons That Are Socially Legitimate

The account of migrants who enter with permits that are at odds with their motivations might cause apprehension or disapproval. If half of the people who are given residence permits based on family ties cite work opportunities as their main motivation, are they entering on deceptive terms?

Regardless of the circumstances that might produce these outcomes, the reproach, or potential for it, is interesting in its own right. Reasons for migration are morally codified by migrants and their families, by states, and by the societies that migrants leave and enter. The sway of moralities, combined with the tenuousness of motivations, has great implications for how the reasons for migration are presented and understood.

Migration researchers have primarily examined the intersection of immigration policy and culturally specific moralities in two areas: assessments of “proper marriages” versus “sham marriages,” and notions of refugee deservingness and “bogus refugees” (e.g., Andrikopoulos 2021; Bonjour and de Hart 2013; D’Aoust 2017; Palillo 2022; Vandevoordt and Verschraegen 2019). In both cases, there is a normative ordering of thoughts and feelings that “ought to” motivate migration, and it plays out in the context of fears that the immigration system is abused.

But the moralities of why people migrate is a broader issue, and not necessarily linked to policy. A vignette from village in Honduras, related by Reichman (2011, 548–549) illustrates. Verónica, a mother of two, had made a deal with a *coyote* (smuggler) and was about to migrate to the United States. She explained her decision in ways that aligned with standard social-scientific understandings of South-North migration: coffee prices had fallen, her family had lost their livelihood, she had struggled to make ends meet as a factory worker, and now she wanted to travel North and earn enough money to pay off debts and recreate a decent life for her family upon return. But a villager who heard that Verónica was about to leave had a different explanation: “they wasted the family fortune. They humiliated their family and had no choice but to leave. How could they stay with such shame?”

This gossipy tale reflected a broader pattern, Reichman notes, whereby migrants themselves pointed to economic compulsion while others in the village would see the migration decision as a personal choice, or the result of character flaws or moral weakness. The contrasting representations of Verónica's migration are telling, in that the apparent facts of the story are largely the same, while the essential “why” of migration is different.

In the context of family migration, the “why” is generally assumed to be that family members want to be together. Kofman, Buhr, and Fonseca (2022, 137) outline the scope of family migration:

To move for family reasons may encompass an array of different kinds of migration trajectories, from the adoption of a foreign child to family members accompanying migrant workers or refugees, as well as people forming new family units with host country residents. Yet, the primary form of family migration remains family reuniﬁcation: when family members reunite with those who migrated previously.

This is all true, of course, but occludes the less socially legitimate forms of migration for family reasons. Family can be a push factor as well as a pull factor.

Once again, the THEMIS survey can illustrate. In addition to asking for reasons for moving to the destination, as discussed above, we asked respondents about six potential reasons for leaving their country of origin. In all three groups—Brazilians, Moroccans, and Ukrainians—between 10% and 20% said that “family difficulties” were an important motivation for leaving.^3^ The figures were slightly higher for women than for men, but not much.

Ethnographic research from various parts of the world has touched upon the kinds of dynamics that might be at work, though they are rarely a major theme. Several studies on female migration in south-east Asia have shown how migration for work is compliant with the role of the “dutiful daughter” (by way of remitting to the family) and at the same time allows for escaping the everyday subordination to parents and other elders and leading more independent lives (Constable 2007; Mills 1999; Rigg 2007). Here we see two compatible motivations for migration: one that conforms to social norms and one that challenges them.

When migration is motivated by marriage, the motivations are far more diverse than spouses wishing to live together. First, migration can be part of women's strategies for staving off an unwanted arranged marriage, delaying marriage, improving their prospects on the marriage market, or providing a foundation for greater autonomy in marriage (Belloni 2020; Engebretsen et al. 2020; Khoo and Yeoh 2017).

Migration can also be a strategy for leaving abusive or unhappy relationships. In fact, ethnographers have observed this both in societies where the threshold for divorce is high, such as the Philippines (Madianou 2012; Parreñas 2015), and in societies where conjugal relations are often informal and unstable, such as Cape Verde (Åkesson 2004). Tanja Bastia (2013) also relates, from Bolivia, stories of women who have used migration to leverage power over their husbands and mend troubled relationships.

Beyond emotional strain in close relationship, family and kin can be a push factor because of economic expectations. In West and Central Africa, in particular, ethnographers have examined how entrepreneurial aspirations can be stunted by redistributive norms. Migration can ease this balancing act by allowing for individual accumulation and investment without an outright rejection of social obligations (Doevenspeck 2011; Hernández-Carretero 2015; Whitehouse 2013).

In a striking account, Bruce Whitehouse (2013) describes how thousands of migrant entrepreneurs moved from West Africa to Brazzaville, in the Republic of Congo, at a time when their destination was dubbed “the world's worst city.” The migrants relocated to *worse* economic and security conditions because these burdens are outweighed by the benefit of distance to kin. In other words, the motivation for migration has everything to do with family dynamics, but is completely detached from the notion of “family migration.”

When people are socialized into explaining migration in certain ways, it is not only a question of legitimacy, but also a source of convenience. People do get asked about their reasons for migration, and it can be a difficult question to answer. The accessibility of explanations can therefore outweigh their importance. In her study of Brazilians in Ireland, Karine Dalsin (2016, 172) fond that they tended to have a “ready-made answer […] at the tip of their tongue.” This was “to learn English” which was almost always the response in ordinary conversation. However, interviews revealed that language skills often played a minor role while other issues provided fuller views of motivations.

The notion of a ready-made formulation of motives can be relevant even before migration. In cultures of migration, the act of migrating is a social construct embedded with specific meanings, and often talked about with specific phrases. In Cape Verde, for instance, migrating is typically linked to the notion of “making a life.” Åkesson (2004) exemplifies with the case of a woman on the cusp of migrating alone, who says “I have to leave in order to make my life” and seems to leverage the ready-made phrase “as a protective shield against her children's tears, her elderly father's silence, and the sulky attitude of her boyfriend.” Alioua (2008, 710) notes that African migrants more generally “use the formula ‘I am going to look for my life’ [*je vais chercher ma vie*] to explain, and at the same time legitimize, their migration.”

The dimensions of legitimacy and convenience can help understand why reasons for migration are represented in specific ways. This does not mean concealment of an underlying “true” reason, but rather points to the malleability of explanation and the need to treat all accounts with openness and curiosity.

### 3. Because the Sum of Push and Pull Factors Is in Favor of Migration

People migrate because there are push factors at the origin, pull factors at the destination, or both. This is a largely self-evident statement that nevertheless triggers unease. For the past three decades, “push–pull models” have served as a strawman in migration theory, a foil for claiming a more nuanced or sophisticated approach.^4^ However, there is no established “push–pull theory” and the sources that are most often cited—Harris and Todaro (1970), Lee (1966), Todaro (1969), and Ravenstein (1885)—never use “push” and “pull” other than in passing, if at all. In other words, “push–pull theory” is a bit of a phantasm beyond the commonsensical idea presented at the beginning of this paragraph.

The closest we come to a coherent theory with push–pull elements is Everett Lee's (1966) “theory of migration.” It is most commonly associated with the succinct visualization of positive, negative and neutral factors at the origin and destination, separated by intervening obstacles. The original figure was labeled “see text for explanation” and the text does indeed cover many aspects that are often lost.

Already in the 1970s, Lee's model was misleadingly described as “the push–pull model” (e.g., Ritchey 1976; Uhlenberg 1973). But, in fact, Lee's approach holds up against many of the criticisms leveled at push-pull theory. As de Haas (2021) rightly points out, the semantics of “push” and “pull” obscure the agency of migrants and render them as pawns that are moved by external forces. Yet, in Lee's terminology, there were simply “factors associated with” the origin and destination, respectively, which potential migrants might value positively or negatively. And while “push–pull theory” is commonly criticized for privileging economic forces and ignoring social norms and expectations, Lee primarily uses non-economic examples and emphasizes that it is individual priorities and perceptions, rather than external facts that matter.

A different criticism of “push–pull theory,” voiced by Skeldon (1990, 2021, 36), is that it falls short of being a theory. “At best,” Skeldon writes, “pushes and pulls provide a systematic description, even a listing, of the factors […] that give rise to migration.” In fact, he continues, they are “more likely to be associated with, rather than directly causing” migration.

In my opinion, this is too dismissive. “Theory” in the social sciences can have diverse productive purposes beyond postulating testable causality (Sandberg and Alvesson 2021). Pragmatically, we can simply ask whether Lee's theory of migration helps understand why people migrate—and, by extension, why others stay.

We have already seen how Lee's concepts help order a messy or perplexing reality, in Bruce Whitehouse's (2013) account of migration to Brazzaville. He points out the utter absence of economic pull factors, as well as the significant “intervening obstacles” (to use Lee's terminology) in the form of a 3000 km overland journey. The decisive counteracting force is social networks working as a push factor. Lee's model could take us further towards examining whether this migration appealed specifically to individuals who felt they had a potential for upward mobility, and therefore feared the leveling effect of redistributive norms.

In the midst of push–pull skepticism, Van Hear, Bakewell, and Long (2018) made the case for an approach that they termed “push–pull-plus.” “While acknowledging the critiques of analysts who view push–pull models as too simplistic and determinist” they wrote “we still find some merit in the simple notion of push–pull, with its intuitive and empirically grounded idea that structural forces shape migration processes” (2018, 928). Their proposed *driver complexes* add valuable process-oriented dimensions that are, indeed, missing from original work associated with a push–pull approach.

Yet, the notion that push–pull approaches emphasize structural forces is perhaps misleading, regardless of whether this is seen as a weakness or a strength. In line with Lee's (1966) arguments, we can examine how and why people both *perceive* and *evaluate* factors differently, and make migration decisions accordingly. But using “push–pull” as inspiration for such an analysis is obstructed by a glaring selectivity: *push* and *pull* factors are merely two of the four types of factors that matter.

*Retain* and *repel* factors are just as important. They are the pluses at the origin and minuses at the destination, which motivate people to stay. Kerilyn Schewel (2020) made this point as part of her effort to make explanations of immobility more prominent in migration studies. In other words, an obvious step toward discarding the push–pull strawman is to consider the full picture: push–pull–retain–repel, all of which are socially conditioned individual perceptions as much as structural forces.

Lee's basic idea is that the perceptions can be added up to give potential migrants a sense of the anticipated gains of migrating. But even if they are positive, they might not be enough to overcome what Lee calls the “intervening obstacles” to migration. For instance, a dangerous journey may or may not be considered a price worth paying for anticipated safety at the destination.

Here we start encountering a fundamental challenge, even with more nuanced “push–pull–retain–repel” thinking that extends to non-economic factors and obstacles. In Lee's vision, these elements are all *fungible*. In other words, the intervening obstacles can be offset by anticipated gains because everything is abstractly converted to the same currency, so to speak. But in a world of barriers to migration, crossing borders does not only depend on willingness to accept the cost. This realization prompted a new turn in migration theory, separating the aspiration and the ability to migrate.

### 4. Because They Have the Aspiration and the Ability to Do So

People migrate because they see leaving as preferable to staying *and* have the ability to act on this assessment. This tenet explains why most prospective migrants *don’t* move. For every international migrant, there are roughly three others who say they would want to migrate permanently, but are still in their own country.^5^

The question “why do people migrate?” should therefore be broken down into separate inquiries into (a) the aspiration (or desire or plan) to migrate, and (b) the ability (or capability or capacity) to migrate. This is the essence of the aspiration/ability model (Carling 2002). It offers, for instance, a simple explanation why migration tends to be most common at the middle levels of socio-economic status: the wealthiest groups lack the aspiration to leave; the poorest groups lack the ability to leave; the people in the middle are most likely to have both.

The past decades have seen a swell of research into the first question implied by the aspiration/ability model: “why do people aspire to migrate?” Extensive new survey data have shown, among other things, that low life satisfaction, experiences of corruption, and high levels of educational attainment are among the factors that tend to stimulate migration aspirations (Aslany et al. 2021).

The separation of aspiration and ability suggests that the “reasons” for migration can be very real also in the absence of people crossing borders. For instance, imagine a society where corruption spurs widespread migration aspirations, but produces only a trickle of migration because most people lack the ability to leave. Those who do migrate might even be people who stood to benefit from corruption. Pursuing the question “why do people migrate?” by asking the migrants would give a distorted image of the processes at work. Examining migration aspirations is therefore not merely a second-best alternative to examining migration behavior (Carling 2019; Carling et al. 2023).

Research on the ability to migrate is clearly more challenging. Because how do we know whether people are able to migrate unless they actually do so? The aspiration/ability model sidesteps this challenge by only considering what we might call “revealed ability,” or ability that is manifested in migrating. People who expressed migration aspirations and have not migrated are assumed to lack the ability. And for people who do not aspire to migrate, ability is deemed irrelevant.

In the extension of the aspiration/ability model that he called the “aspiration–capabilities framework” Hein de Haas (2014, 2021) took a different route, incorporating Amartya Sen's (1999) capabilities approach. At the core of this approach, is the idea that expanding freedoms to choose is a defining feature of development. Consequently, “migration capabilities” are valued regardless of migration aspirations.

This is a normatively meaningful stance, but poses some theoretical and methodological challenges. De Haas (2021, 20) defines migration capabilities as “the ability to decide where to live, including the option to stay at home” and points out that they can be enjoyed “without ever using them.” The question, then, is how we can assert that people have the “capabilities” to migrate if they have never considered it, aspired to do so, or made any attempt. Apart from the empirical challenge, we might wonder, ontologically, whether migration capabilities in this sense truly “exist.”

Analytical approaches to both aspiration and ability (or capabilities) are continuously developing, but the separation of the two is already a decisive step toward understanding.

### 5. Because an Opportunity Presents Itself

People migrate because they encounter an opportunity, which might be unforeseen. This idea is both an extension and a corrective to the theories discussed in the previous section. The logic of an opportunity that appears grates against the notion of having or not having migration capabilities. It also contrasts with the sequential logic of the aspiration/ability model, which assumes that migration aspirations come first, and conversion into actual migration follows.

The possibility of more complex interactions was part of the original formulation of the aspiration/ability model, but more as an afterthought. I reasoned that, in an environment where the ability to migrate is scarce and coveted, someone who does not have clear migration aspirations, but is given the chance to go “is likely to feel that they ought to seize the opportunity to emigrate” (Carling 2002, 37). Ten years later, this assumption was confirmed to astonishing degrees in my collaboration with Papa Demba Fall and colleagues in Senegal, where we surveyed young adults about their migration aspirations as part of the EUMAGINE project. More than one third of the people who would *prefer* staying in Senegal said that they would go to Europe if they were offered the necessary papers to do so (Carling et al. 2013). Perhaps such findings call for a broader reconsideration of the role of opportunity in migration theory.^6^

In migration theory “opportunity” primarily features as a structural element, either as “perceived geographical opportunity structures” that shape migration aspirations (de Haas 2021, 17) or as the “migration opportunity structure” of legislation and policy that condition actual migration (Martiniello and Rea 2014, 1089). These structural roles are fundamentally different from how opportunity features, for instance, in the logic of hustling, where opportunities are ephemeral appearances that must be seen and seized by the alert actor (van Stapele 2021).

This non-structural aspect of opportunity has made cameo appearances in studies of migration trajectories, which have examined the dynamics of often unpredictable twists and turns (Mainwaring and Brigden 2016; Paul and Yeoh 2021). The initial departure, too, has been explored as “a sudden, possibly painful decision—one that may have been accelerated by an equally sudden, unmissable opportunity to leave” (Boccagni 2017, 8). A chance encounter, a piece of information, an acquaintance who is suddenly in the right time at the right place—such coincidences can all produce opportunities for migration, and these opportunities might be seized even without preexisting migration aspirations.

These accounts and perspectives challenge the neatness of Lee's theory of migration, the aspiration/ability model, and the aspirations–capabilities framework. They also contrast with psychological models that have inspired migration research, most notably Ajzen's (1991) theory of planned behavior (Kley 2017; Willekens 2017). In light of actual migration dynamics, we also need theory for *unplanned* behavior.

The serendipity of opportunity is central to the notion of *circumstantial migration* which denotes the processes by which migration trajectories and experiences “unfold in unpredictable ways under the influences of coincidence and micro-level context” (Carling and Haugen 2020, 2779). This form of migration creates theoretical obstacles to explaining why people migrate. If it is all serendipitous and idiosyncratic, what is there to investigate? The implication is not, however, to dismiss inquiry, but rather to examine how opportunities emerge, how they are transformed into migration through agility and improvisation, and to allow for the possibility that migration opportunities spur migration aspirations where there were none.

### 6. Either Because They Chose to or Because They are Forced to

People migrate for one of two reasons: out of choice, or being forced. For at least half a century this misguided dichotomy has featured in diverse forms and been thoroughly challenged. But its relevance and consequences are constantly evolving.

Some 30 or 40 years ago, even migration scholars saw forced migration as “beyond the scope of migration theory” (Bakewell 2010, 1690). Today, the dominant view within migration studies is that force/choice is not a meaningful binary for classifying migration or migrants (Erdal and Oeppen 2018; Fussell 2012; King 2002). Rather, volition is understood to play a range of variable roles in migration processes. But this mainstream view does not settle the issue and make the binary irrelevant. The categorical divide between “forced” and “voluntary” migration has proven persistent, also within migration studies, but especially in adjacent research fields, policy circles, and public debate (Hamlin 2021).

The forced/voluntary binary is, to some extent, an inadvertent result of the well-intended embrace of “forced migration” in the 1990s. While refugees are, by definition, outside their own country, the majority people who are displaced by conflict and violence are not. This was a major reason why forced migration emerged as a concept and a field of study (Chimni 2009; Hathaway 2007). The notion of “voluntary migration” seems to have transpired as a logical counterpart, rather than by design.

Despite the diverse categories of forced migrants, the forced/voluntary binary is today reflected most prominently in different understandings of the relationship between “refugees” and “migrants.” Migration scholars would generally agree that refugees are a specific category of migrants. This is also the traditional view of the United Nations, for instance. But there is a competing perspective that sets refugees completely apart. The UN Refugee Agency, UNHCR, contends that refugees are not migrants at all, and that “migration” and “refugee movements” are separate phenomena. This is a long-standing position, but one that the UNHCR has promoted with increasing fervor and success. Since the late 2010s, the UNHCR's annual expenditure on public information and media has exceeded USD 10 million.^7^ The agency has embraced the opportunities of social media and invested in strategic messaging, including the claim that “refugees are not migrants.” Explanations of the difference stress the roles of force and choice, reifying the binary into two mutually exclusive categories of people.

The UNHCR's view recasts “migrants” into an awkward residual category that comprises everyone who moves across a border *except* refugees. This stance helps the UNHCR protect its turf in the struggle for influence among UN agencies, but is damaging to policy, analysis, and protection of people on the move (Carling 2023). The hardline view makes the question “why do people migrate?” irrelevant to refugees. In fact, the messaging often implies that raising such a question is a sign of ignorance or malice.

Yet, there is scope for better, theoretically and empirically grounded, answers to the question “why do refugees migrate?” From the perspective of migration theory, I would argue that the essence of refugee migration is not the absence of choice, but the harrowing nature of the choices.

Examining the interaction of force and choice in the escape from danger or oppression is fraught territory for research (Bakewell 2008, 2010; Stepputat 1999). By studying how refugees exercise agency and make decisions about their migration, researchers run the risk of undermining the case for refugee status—or being accused of doing so. Many scholars have braved this challenge and produced valuable insights (FitzGerald and Arar 2018; Schon 2019; Tarkhanova and Pyrogova 2023), but explanations of migration remain a remarkably marginal topic in refugee and forced migration studies (Erdal, Mjelva, and Tollefsen 2023).

The roles of force and choice in refugee migration are a source of tension also within refugees’ communities of origin. Because in every situation of conflict or oppression, there are some who leave and others who stay. Many stayers would have wanted to leave, but lack the ability to do so; others stay by choice. The stayers can have their own answers to the question “why do refugees migrate?”

“I could detect widespread resentment towards refugees who escaped during the war” wrote Nadje Al-Ali (2002, 258) after visiting Bosnia-Herzegovina. Like most refugee studies scholars, she had worked with refugees in their countries of settlement. It was only after going to their communities of origin, she wrote, “that I started to grasp the tensions and conflicts between refugees and those ‘who stayed behind.'” Indirectly, the tensions reflected animosity towards the choice that male refugees, in particular, had made in leaving. Because of that choice, they were branded “cowards” and “deserters.” Moreover, refugees were assumed to be accumulating wealth abroad while those who had never left grappled with economic crisis. More recently, after the 2022 Russian invasion of Ukraine, men aged 18–60 were not only legally prohibited from leaving Ukraine, but also subject to moral condemnation and accusations of treason (Mickelsson 2023).

It is striking that, in each of the two forms of tensions I have discussed, the question of “why refugees migrate?” causes animosity on one side of larger conflicts. In research and policy, actors who all sympathize with refugees are divided over whether this is a legitimate question to ask. And populations who are victims of the same aggressors are divided over whether or not fleeing is an act of selfishness and cowardice.

Renderings of male refugees as cowards raise many questions about the gendering of deservingness and the hierarchical ranking of loyalties to nation and family. Moreover, this narrative is seized upon by xenophobic forces in destination countries and compounds the hardship of refugees (Glăveanu, de Saint-Laurent, and Literat 2018; Rettberg and Gajjala 2015).

Explorations of refugee agency and decision-making are, overall, the most politically and ethically treacherous realm of responses to “why do people migrate?” It is all the more important to support and build upon the work of scholars who have ventured into this terrain in conscientious and perceptive ways.

Taking a step back from refugee politics, the role of force or coercion merits attention also in other forms of migration. For instance, Matthew Gibney (2013) pointedly asks why the study of forced migration generally ignores deportation. The absence of choice can also be stark for children and adolescents whose migration is orchestrated by parents. In summary, conventional uses of the force/choice binary rarely give satisfactory answers to why people migrate.

### 7. Because They See Migration as Either Intrinsically or Instrumentally Valuable

Despite disagreements over how to approach refugee migration, there is a shared understanding that refugees leave a situation of danger or oppression to obtain safety elsewhere. Migration is a *means to an end*. The same is true for migration that is an instrumental step toward livelihoods, education, or family life, for instance.

Seeing migration as instrumental is a meaningful approach to explaining most migration, but not all. Migration theory must also allow for the possibility that people see migration as *intrinsically valuable,* as an end in its own right rather than a means to something else.

The intrinsic/instrumental distinction is a recent development in the long history of migration theory, first introduced about a decade ago (Carling 2014; de Haas 2014). It does not lend itself easily to empirical application, but it is a potent analytical device.

The distinction between the intrinsic and the instrumental lies in closer examination of responses to “why do people migrate?” If the motivation is something that could, hypothetically, be obtained in other ways, migration has instrumental value. For instance, the safety sought by refugees could also be obtained by the cessation of conflict or repression, and if the livelihoods sought by labor migrants were available locally, they might not have migrated.

If people migrate—or aspire to migrate—due to the instrumental value of migration, there are three questions to ask:

1. What is the objective to which migration is a means?
2. What would be alternative means?
3. How are choices made between different means?

As an example, the so-called new-economics of labor migration, posits that a key objective of out-migration (question 1) from rural areas in developing countries is diversification of risk. If some household members work in the city, the argument goes, the vagaries of business cycles and crop yields combine to lessen the fluctuations in the household's total income (Stark and Bloom 1985). An alternative means to the same objective (question 2) would be agricultural insurance. But if such insurance schemes are unavailable, migration is the means that households adopt (question 3).

This example from economics can seem to overplay rationality. But the logic underlying the three questions can also be evident in anthropology, for instance. In Charles Piot's (2010, 77) book based on fieldwork in urban Togo, he has a chapter on the rise of Pentecostalism followed by one on playing the US Diversity Visa Lottery. The latter opens in this way: “If charismatic Christianity represents one response to the current sovereignty crisis, playing the visa lottery is another, providing a complement to the virtual form of surrogation or exit enacted by the Pentecostal.” Although not phrased in terms of strategies and objectives, the underlying analysis of a parallelism is similar.

These contrasting examples illustrate the potential for examining migration as an instrumental step towards a wider range of objectives than the ones implied by standard categories in migration studies and immigration bureaucracies. If we were to attempt a mapping of objectives, other starting points might be more promising. As an example, De Jong and Fawcett (1981) long ago proposed seven “psychologically meaningful clusters [of] values and goals” that motivate migration: wealth, status, comfort, stimulation, autonomy, affiliation, and morality. They have not been widely taken up, but might offer valuable takes on examining what is that people seek when they migrate.

If migration is not a means to an end, but has intrinsic value, the relevant follow-up questions are different, and a bit more abstract:

1. Is the intrinsic value linked to *space* or *place*?
2. How is the thing that has intrinsic value constituted?

Migration is a way of commanding space by being mobile. This can be a source of experience, adventure, or authority that is intrinsic to mobility and cannot be attained by other means. Examples of such valuation in the literature range from the role of migration as a rite of passage (Monsutti 2007; Suso 2020) to the promotion of international mobility in academia (Bauder, Hannan, and Lujan 2016; Robertson 2010). The common theme is that migration or mobility is valued for its expected transformative effect on the individual, and that this effect that is both elusive and irreplaceable (cf. Recchi and Safi 2024).

As these examples show, it is not obvious how migration relates to other forms of mobility. Hence, the second question above: how is the thing that has intrinsic value constituted? If the value of migration lies in the worldliness it bestows on the migrant, for instance, what are the forms of “migration” that has this effect? It is telling that, in West Africa, what social scientists and policy makers call “migration” is consistently referred to as “travelling.” This is not just a matter of linguistics, but reflects the fluidity of the concepts.

One young Ghanaian I have followed over several periods of fieldwork expressed his heartfelt desire to migrate by saying “you know me, I am a traveller!” He did have a plan of working abroad and returning to invest, which reflected the instrumental value of migration, but was also attracted to the intrinsic value of migration, which he sought to embody.

Alternatively the intrinsic value of migration might be tied to a specific place, rather than mobility across space. Migration aspirations can then take the form of geographical yearning. The more the place is valued for its totality of irreplaceable characteristics—as opposed to generic attractions such as a pleasant climate or abundant work—the more migration to that place has intrinsic rather than instrumental value. Erind Pajo (2008) writes about such migration as “territorial fulfilment.”

People do not necessarily yearn for a precise geographical location, but rather, to “place” in the more fluid sense favored by geographers (Cresswell 2015). In societies of origin, migration destinations are often described with particular terms that constitute this sprawling “elsewhere” as a place. Examples include *gurbet* in Turkey (Zırh 2012), *beng* in Côte d’Ivoire (Newell 2012) and *l-brra* in Morocco (Elliot 2021), which all refer to distinctive renderings of the bland “abroad” and affect how migration is imagined.

The intrinsic/instrumental distinction offers compelling opportunities for exploring the question “why do people migrate?”. At the same time, it easily collapses onto itself. If we allow the objectives to be sufficiently abstract, almost all migration can be seen as instrumental. For instance, when Nyberg Sørensen and Finn Stepputat (2001, 313) write that, “experiences of mobility bestow authority on the moving subjects” it resembles my previous description of the intrinsic value of migration, but could also be taken to mean that migration instrumentally serves to obtain authority.

The slipperiness of the intrinsic/instrumental distinction lies in the counterfactual element: would it be possible to obtain the desirable results of migration—such as authority in the example above—by any other means? Often, that's more of a reflection point than an answerable question. Still, it can be leveraged for better understandings of migration motivations, also in direct exchanges with research participants. In the recent FUMI Survey, covering three West African cities, respondents who would prefer to migrate internationally were asked a follow-up question: if they had an income and could live comfortably, would they still prefer to go? Overall, three quarters would then stay, while one quarter would still prefer to go.^8^ For the majority, migration thus seems to be instrumentally valued, and specifically so in economic terms. The minority might value migration instrumentally for other reasons (such as studying abroad, or reuniting with family members) or their motivation might reflect the intrinsic value of migration.

### 8. To Lead a Normal Life

We asked in the FUMI survey about “living comfortably” but the motivation for migration is often more modest. In fact, the quest for normality, decency, and dignity emerges from the ethnographic literature as a powerful driver of migration. Examples include accounts of migration from Albania (Mai 2004), Bulgaria (Manolova 2019), Cuba (Simoni 2019), Poland (Bygnes and Erdal 2017, McGhee, Heath, and Trevena 2012), Serbia (Anđić 2020a, 4), and Tunisia (Mastrangelo 2019). Tanja Bastia's (2013, 171) research in Bolivia is again illustrative. Angela, an interviewee in a poor neighborhood of Cochabamba explained that her ambition for the future was “at least to live like people [*vivir por lo menos como gente*].”^9^ “I don’t expect more,” she said “to always have luxuries. No. Just to live like people.” The only feasible strategy for achieving this, she reasoned, was to work abroad.

For some, as in the case of Angela, the quest for normality is a motivation for working abroad in order to establish a better footing at home. Migration then plays a clearly instrumental role in overcoming the economic “abnormality” of not making ends meet. As an answer to “why do people migrate?” the pursuit of a normal life adds to conventional understandings of economically motivated migration.

First, it establishes a target, as opposed to an open-ended striving for betterment. Even if the target is imprecise, or turns out to be rising along with actual living standards, it shapes how migrants make sense of their own motivations.

Second, the notion of normality draws attention to how this target or threshold is socially constructed. As an illustration, consider how Ania, one of Bygnes and Erdal's (2017, 111) Polish interviewees in Norway, describes the abnormality of life in Poland:

I think the most important issue is about the earnings—that people can’t afford a normal life, that people have to borrow money to live from one payday to another, that thinking about a holiday with your family is almost impossible.

Perhaps a holiday with the family is what Angela, in Bolivia, would consider a “luxury” beyond her ambitions. But speaking as a migrant in Norway, there was nothing luxurious about Ania's desire for at least “thinking about” a family holiday. The parallel is created by both women framing their economic dissatisfaction in terms of normality.

Third, this type of framing lends the economic motivation for migration a moral and political dimension. Angela's choice of words invites the reaction “of course you deserve to live like people!” The framing of normality also gives the economic motive a sense of urgency without being about escape from destitution.

Beyond the instrumental value of migration for securing a “normal” standard of living, a quest for normality could also motivate migration in order to establish a life abroad, for more expansive reasons. Valerio Simoni (2019, 2) summarizes from fieldwork in Cuba that “life ‘abroad’ appeared as having some measure of ‘normality’: a normal life, a normal job, a normal family, a normal friendship, and love.” The counterpart of abnormality at home thus seems deep and totalizing—reminiscent of the image of Colombia as a sick, rabid dog.

Whether abnormality is felt as societal feature or individual circumstance, it stands out as a motivation that seems pervasive, powerful, and largely overlooked in the migration literature. In fact, it has even been used to define what migration is *not* about. The economist Paul Collier (2016), who wrote the widely debated book *Refuge* together with Alexander Betts, mistakenly argued that “refugees are not migrants” because their aspiration is simply “to find a normal life.”

While many prospective migrants see migration as a *means to obtain* a normal life, others see it as *part of* a normal life. This too has a bearing on how to approach the question “why do people migrate?” In short, the desire for explanation might be misplaced. As an illustration, a British colleague of mine who has lived in Norway for several years told me that she often gets asked “what are you doing here?” She was initially perplexed by the question, but now routinely just answers “I live here.” Even if there is no hostility in the request for her justifying her presence, it reflects the weight of the view that there must be a reason for living in a country which is not where you were born.

Research methodology should accommodate diversity in the salience of reasons for migration, as something that requires explanation. In a survey of Polish migrants in Western Europe, Renee Luthra, Platt, and Salamońska (2018) and her colleagues astutely included “just because” as a response option to the question “why did you move?” It is illuminating to see this as distinct form of motivation, rather than relegating it to response options such as “other” or “don’t know.” In total 11% of the sample said that they migrated “just because.”

## Alternative Takes and Cascading Explanations

The preceding eight sections laid out alternative ways of approaching the question “why do people migrate?”. My aim was to make the case for the potency of this question and use it to challenge and complement prevailing explanations.

The eight ways of answering “why do people migrate?” are summarized in Table 3. The table's second column specifies what each approach does, its *function* in informing migration research. The final column spells out implications that emanate from the discussion of each approach. As the table illustrates, the eight approaches are not competing theories, but complementary takes on the question, which leverage it for diverse insights.

**Table 3.** Implications of the Eight Responses to “Why Do People Migrate?.”

Response	Function	Implications
1. For the reasons under which they are admitted as immigrants	Exposes an entrenched misconception	Grounds for admission should not be conflated with motives for migration. General thinking about reasons for migration subtly reflects bureaucratic categories.
2. For reasons that are socially legitimate	Underscores mechanisms of representation	Reasons for migration vary in salience because of their social, moral, and political dimension. Formulations of reasons for migration are social constructions, and some are more cognitively accessible than others.
3. Because the sum of push and pull factors is in favor of migration	Amends the conception of a disparaged approach	People’s individual perceptions of positive and negative factors associated with the origin and destination are enlightening for understanding migration aspirations. Explanations lie not only in push and pull factors, but also in retain and repel factors. “Push–pull theory” is largely a stereotyped foil and should not stigmatize sensible references to push and pull factors that motivate migration.
4. Because they have the aspiration and the ability to do so	Makes an analytical distinction	The question “why do people migrate?” easily conflates the separate roles of aspiration and ability. There is a distinction between ability, which is revealed through migration, and capabilities, which are seen to exist independently of migration aspirations or attempts.
5. Because an opportunity presents itself	Emphasizes often-overlooked explanation	Migration can be caused by a logic of seeing and seizing opportunities, as opposed to making specific plans. The reason for migration might simply be that it became possible, regardless of preexisting aspirations.
6. Either because they chose to or because they are forced to	Exposes an entrenched misconception	The false binary of voluntary and forced migration should not be upheld inadvertently or implicitly through terminology Explanations of refugee migration are relatively marginal in the literature, partly because they are controversial and sensitive.
7. Because they see migration as either intrinsically or instrumentally valuable	Makes an analytical distinction	Seeing migration as an instrumental means to an end allows for querying the nature of the objective and the existence of alternative means. Seeing migration as intrinsically valuable disrupts conventional explanations and inspires new questions.
8. To lead a normal life	Emphasizes often-overlooked explanation	When migration is motivated by a quest for a normal life, explanations must partly be sought in constructions of normality. Migration can be understood as part of normality and therefore defy specific explanation.

The question yields radically different answers not only because it is open to such diverse approaches, but also because “why” questions can cascade: every answer can be followed by another “why?” This sequential questioning is familiar to anyone with young children. It has also been promoted as a management and quality improvement tool known as the “five whys,” with five being a somewhat arbitrary number. The reasoning is that successive whys provide a path from the outcome of interest (e.g., medication given to wrong patient) to a fundamental cause, which is where remedial measures should be focused (Berger 2014).

**Figure 1.**
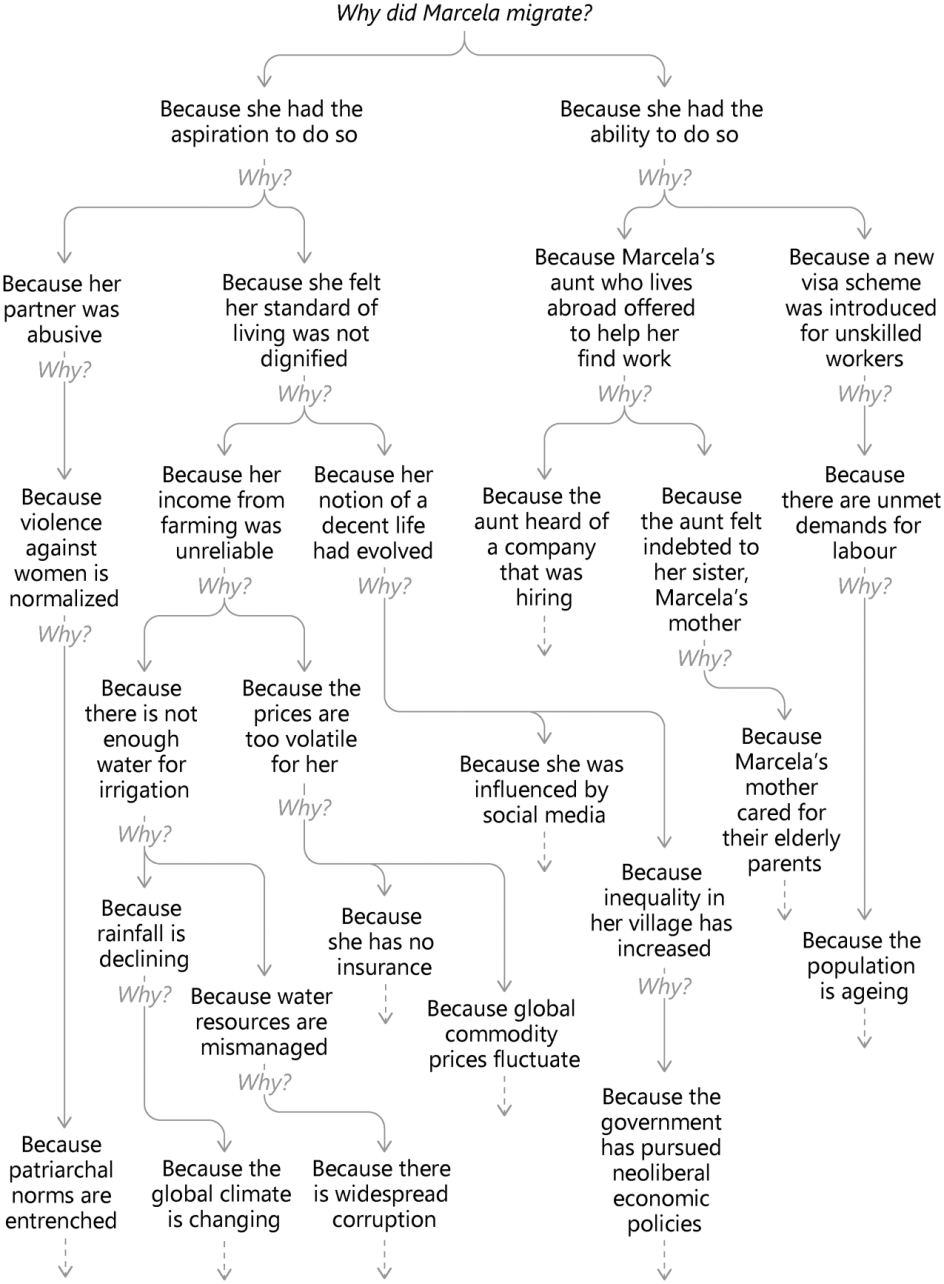
Cascading explanations of migration.

*Note:* Dashed arrows indicate potential extensions.

Without seeing migration as a problem to be fixed, we can imagine similar interrogative sequences towards more overarching explanations of why people migrate. In this vein, Figure 1 shows a cascade of answers to why Marcela, a hypothetical migrant, migrated. The cascade is structured by the initial distinction between aspiration and ability (take 4), which is an analytical choice, and a gateway to exploring complexity. Starting with push and pull factors (take 3) or intrinsic versus instrumental value (take 7) could have yielded different, but also explorative cascades. By contrast, focusing on Marcela's quest for a normal life (take 8) or her seizing of an opportunity (take 5) would have guided attention to specific reasons that are part of the fuller picture presented in Figure 1, but frequently disregarded. Simply asserting that Marcela migrated out of choice (take 6) or to seek employment (take 1) would not be wrong but would be partial truths that obscured the role of her abusive partner, for instance. Finally, attentiveness to social legitimacy (take 2) stimulates critical curiosity about the answers that might be given—by Marcela herself, people around her, and outside observers.

Indeed, the analytical choices that are evident in Figure 1 reflect the potential sway of social legitimacy also in academic and political environments. Do we attribute the water shortages that hampered Marcela's farming to decreasing rainfall or to mismanagement of water resources? And are her rising material ambitions driven by social media or by growing socio-economic inequalities? These are partly empirical questions, but not only.

As we follow the cascading explanations in Figure 1 we approach momentous structural factors such as population ageing, neoliberal policies, climate change, and patriarchy. Some scholars see the task of social scientists as connecting these kinds of forces to observable outcomes for individuals, while others focus on the factors that are closer to behavior. So, even the prioritized tier of the cascade is a matter of choice.

All the sequences of whys that led to a fundamental factor deep down in the cascade are plausible, but each one provides only a blinkered view of the complex causal pathways that explain Marcela's migration. This is a fundamental problem with seeking explanation through sequential whys (Card 2017). The role of climate change is a case in point: there's little doubt that climate change affects migration, but it is hard to pinpoint individual “climate migrants.”

In the face of such complexity and room for different explanatory strategies, there's first and foremost a need for reflexive awareness of available alternatives and their implications—implications for scientific understanding, for policy and politics, and for the ethics of research. This is where the eight takes on “Why do people migrate?” can be valuable. What are we losing by adopting a specific take? What would change if we adopt another one?

In empirical research, such exploratory reflection must coexist with concerns about epistemology (what is knowable) and methodology (how to obtain knowledge). In the next and final section of the paper, I pursue an element of closure by addressing where the insights leave us as migration researchers, and what they imply for research practice.

## Methods in Pursuit of Why

“If you want to know why a person migrated, why not just ask them?” This proposition, Gillespie, Mulder, and Eggleston (2021, 2) point out, is an intuitive starting point for data collection, yet turns out to be a can of worms. Rather than seeing these methodological and conceptual challenges merely as frustration, we can leverage them to ground the preceding theoretical discussions in methodological choices.

### Reasons, Motivations, and Causes

As researchers, we should critically reflect on which kinds of answers we seek. The question “why do people migrate?” is stunningly open in this respect. From an analytical perspective, it invites answers in the form of *reasons for human action*. But “reasons” and “human action” are both tricky concepts that require further probing before they can be used in empirical research.

A rudimentary take-away from contemporary philosophy of action is that *justifying* reasons are different from *motivating* or *explanatory* reasons (Alvarez 2017; Sandis 2009). For instance, there may be many reasons that justify a woman's migration from Honduras toward the United States—reasons in the form of facts that render migrating a good, appropriate, or sensible thing to do. But perhaps only one of these reasons actually motivated her to act. The remaining reasons are still relevant as social and political constructions that form the context for her decision and her representation of it.

Reasons for action are, to a large extent, also obscure for the actors. When a migrant gives a reason why she migrated, she is, according to the arguments of the philosopher Hacker (2009, 87), simply endorsing a certain kind of description of her migration and taking responsibility for it under that description. “It is bizarre” he argues “to suppose that reasons are causes.”

In the migration literature, “causes of migration” have gradually yielded to “drivers of migration” as the preferred term (Carling and Collins 2018), perhaps because it has fewer epistemological complications. But drivers, as well as causes and determinants, shift the focus away from individual agency towards factors that, in combination, shape the size and direction of migration flows. Being male, for instance, is a powerful determinant of migration, but hardly offers a meaningful answer to “why do people migrate?” Instead, this question calls for answers that provide reasons both at the individual and the aggregate level. And if we understand reasons to include motivations, it seems necessary to collect data *from* individuals, rather than just about them.

### Asking, Answering, and Analyzing

Survey research is sometimes regarded skeptically as a box-ticking exercise that eschews the nuances of people's perceptions and experiences. But precisely because the format is so constraining, *developing* a survey can stimulate constructive confrontation with theoretical challenges. If a survey includes the question “why did you migrate?” deciding on appropriate response options is a hugely challenging theoretical and methodological exercise. It is difficult, not least, because reasons do not pre-exist as discrete phenomena. Therefore, when reasons are listed as response options, they often include items that might be hard for respondents to distinguish, such as discrimination versus political persecution versus religious persecution (Bilsborrow et al. 1997) or better quality of life versus improve future for family, versus peace/absence of war (Schellenberg and Maheux 2007). It might be sensible to allow for multiple responses, but doing so does not resolve the problem of fuzzy relations between the options, unless the overlaps are a deliberate part of the design (Spaan, Henkens, and Kalmijn 2023).^10^ Similarly allowing for “other” reasons to be specified can seem like a safety valve but end up serving mainly to document the weaknesses of the predefined options.^11^ The main implication for survey researchers is that formulating good response options to such a question requires a substantial investment in understanding the empirical context and reflecting on the theoretical and methodological implications of the choices. Also, as previously mentioned, research on international migration can draw inspiration from surveys on internal migration, that often cover a broader range of less bureaucratically determined reasons.

An alluring alternative to formulating response options, also in a survey is to allow respondents to formulate an answer in their own words. Doing so can yield data that is unwieldy and challenging to analyze, but potentially rich in insights (Gillespie, Mulder, and Eggleston 2021). A limitation, of course, is that many such responses will be elliptical, ambiguous, or otherwise hard to use.

Leaving the survey format altogether and doing qualitative interviews offers respite, with possibilities for probing and follow-up questions. However, reasons for migration can remain obscure. The “ready-made answers” of Brazilians in Ireland, referred to earlier, obscured a deeper understanding of the reasons for migration in Karine Dalsin's (2016) interviews. Her methodological response was to ask about process rather than outcome, for instance exploring why an individual migrated at a specific time, rather than before or later. “In most cases,” she notes

interviewees did not provide condensed answers about why they migrated; instead, they delivered insights piecemeal, each adding more information. Indeed, sometimes the most telling details emerged when they spoke about other topics. (Dalsin 2016, 173)

This experience shows the value of qualitative methods in eliciting nuance in reasons for migration. Probing for finer distinctions in a survey, by contrast, can back-fire when the kind of answers sought by researchers do not resonate with respondents. For instance, a survey of internal migrants that, for theoretically good reasons, asked separately about reasons for *moving from* the origin and reason for *moving to* the destination produced a slew of annoyed comments such as “how many times can you ask the same question before it gets ridiculous?” (Gillespie, Mulder, and Eggleston 2021, 8).

### Aspiration, Ability, and Explanation

When we ask “why do people migrate?” it is apparently the “why” that is the most challenging component while the “human action”—migration—is comparatively straightforward. But human action refers to “the kinds of things human beings can do or refrain from doing at will” (Hacker 2009, 75), and this is hardly a fitting description for migration, especially international migration from The Global South. In short, people might not be in a position to “decide” to migrate any more than they can decide to win the lottery.

In practice the question “why do people migrate?,” therefore, tends to be about migration aspirations. This is clear from the typical response options in surveys and the kinds of answers given in qualitative research. So, a more precise question would be “what made you conclude that migrating would be better than staying?” And this question could be asked in almost identical ways *before* migration. People who express migration aspirations can be asked what *makes them* conclude that migrating would be better than staying. Regardless of the phrasing and format, it is a momentous fact that the essence of “why did you migrate?” is relevant both before and after migration. This attests to the value of the burgeoning research on migration aspirations for understanding why people migrate.

The more complicated aspect of “why” has to do with ability or opportunity, rather than with aspiration or motivation. If a man and a woman experience the same violence and insecurity and both want to escape, but only the woman succeeds, how can we pursue an answer to *why* she migrated? The violence was a motivating reason, but hardly a satisfactory explanation, since it says nothing about why one person stayed while the other left. The theoretical and methodological take-away is that sound explanations of migration must also be explanations of non-migration.

### “Why?” as a Beacon

Perhaps it is true that “why?” is not a scientific question. Indeed, “why do people migrate?” defies a single satisfactory answer. But the question's indeterminate nature makes it a guiding light for research that navigates a diversity of perspectives with humility and curiosity.

How do prospective migrants make sense of their own migration aspirations? What are migrants’ retrospective reasonings for moving? How and why do others represent reasons for migration in particular ways? Such questions, derived from the nexus of “why?,” bridge theoretical, methodological, and political dimensions of migration and hold the promise of new insights in future research.

## Notes

1 Personal communication, seminar at Maastricht University, 2016.

2 This applies to intra-European migrants. Non-European immigrants, who are subjected to more onerous immigration procedures, are assigned a “motive” for migration on the basis of the type of permit they were given.

3 *N* = 2841; weighted to reflect sampling design. Exact figures are 18% among Brazilians, 11% among Moroccans, and 13% among Ukrainians.

4 Garelli and Tazzioli (2021, 387) similarly describe push–pull theory as a “long-time foil for critical migration studies.” The criticisms that are discussed, and partly dismissed, in this section have been made by de Haas (2021), O’Reilly (2023), Schewel (2015) and others, as parts of discussions with many other valid contributions.

5 Survey data from the Gallup World Poll indicate a total of 900 million people wishing to migrate in 2021 (Pugliese and Ray 2023) compared with an estimated international migrant stock of just under 300 million.

6 I am grateful to Tone Sommerfelt for our joint ongoing work exploring the theoretical potential of “opportunity.” See https://prio.org/fumi.

7 Budget and expenditure documents including UNGA 2023 “Programme budget for 2024 of the Office of the United Nations High Commissioner for Refugees” (A/AC.96/74/5) and the UNHCR Global Appeal 2020–2021.

8 Future Migration as Present Fact (FUMI) Survey, carried out in Serekunda (The Gambia), Tema (Ghana), and São Vicente (Cape Verde). *N* = 2515 for this variable. See prio.org/fumi for details.

9 Spanish original obtained from the author.

10 If, for instance, one cluster of reasons is differentiated as three overlapping options while another cluster is represented as one broad option, the former might attract more responses for this reason alone.

11 The full-text specifications may not be possible to categorize in a comparable manner. And if one or more specific reasons stand out in the full-text specifications, it is likely that it would have attracted a greater number of responses if it were included in the original list.
